# Predictive Value of the Esterman Visual Field Test on the Outcome of the On-Road Driving Test

**DOI:** 10.1167/tvst.11.3.20

**Published:** 2022-03-17

**Authors:** Yasmin Faraji, Marian T. Tan-Burghouwt, Ruud A. Bredewoud, Ruth M. A. van Nispen, Laurentius J. (René) van Rijn

**Affiliations:** 1 Amsterdam UMC, Ophthalmology, Amsterdam Public Health, Vrije Universiteit, Amsterdam, The Netherlands; 2 CBR, Rijswijk, The Netherlands; 3 Department of Ophthalmology, Onze Lieve Vrouwe Gasthuis, Amsterdam, The Netherlands

**Keywords:** visual field defects, driving, on-road driving test, glaucoma, Esterman visual field

## Abstract

**Purpose:**

As the prevalence of age-related visual field disorders and the number of older drivers are rising, clear criteria on visual field requirements for driving are important. This article explores the predictive value of the Esterman visual field in relation to the outcome of an on-road driving test.

**Methods:**

A retrospective chart review was performed for driver's license applicants who, based on their visual field, performed an on-road driving test. Cases (*N* = 101) with a failed on-road driving test were matched with 101 controls with a passed outcome. The Esterman visual field was divided in regions, and the number of points missed per region was counted. Logistic regression models and receiver operating characteristic (ROC) curves were computed for each region.

**Results:**

Most regions presented a significantly increased odds for failing the driving test when more points were missed. The odds ratio for the whole visual field was 2.52 (95% confidence interval, 1.53–4.14, *P* < 0.001) for all the participants. However, ROC curves failed to reveal distinct fail–pass criteria based on the number of points missed, as revealed by a large amount of overlap between cases and controls.

**Conclusions:**

These findings confirm the relation between visual field damage and impaired driving performance. However, the Esterman visual field results were not conclusive for predicting the driving performance of the individual driver with visual field defects.

**Translational Relevance:**

In our group of participants, the number of on-road driving tests cannot be further reduced by a more detailed definition of fail–pass criteria, based on the Esterman visual field test.

## Introduction

A driver's vision and visual field are vital for the assessment of driving performance in order to ensure public and road safety.^1^ Hence, in most countries, visual field criteria apply for obtaining and retaining a driver's license. Research on this topic is becoming increasingly important, as the prevalence of age-related visual field disorders and the number of older drivers are rising.^2^

Many studies report on the relation between visual field defects and driving performance. Yet, the available evidence remains inconclusive.^3^ Some studies find that visual field impairment is associated with an increased risk of being involved in a motor vehicle collision.^4^^–^^7^ Other studies do not show such a correlation.^8^^–^^11^

This discrepancy may be explained by the fact that driving is a highly visual task involving visual sensory functions, such as spatial resolution, contrast sensitivity, and light sensitivity. Controlling a vehicle takes place in a visually cluttered environment with many distractions and involves the simultaneous use of central and peripheral vision, cognitive functions that a conventional visual field test does not measure.^12^ Another aspect that is not measured by visual field tests is compensatory head and eye movements.^13^^,^^14^ Some drivers may be better adapted to their visual field defect than others by better scanning toward blind areas.^15^ Even some people with complete hemianopia have demonstrated to be safe drivers.^16^^,^^17^

Presently, formal visual field testing is still the first step for acquiring a driver's license when there are suspected visual field abnormalities. In The Netherlands, the legislation mandates a minimum horizontal visual field of 120 degrees and a minimum left/right reach of 50 degrees. The vertical visual field must extend to 20 degrees above and below. Within the central 20 degrees (radius), no points on the Esterman may be missed (apart from the physiologic blind spot in monocular drivers), since a single missed point may indicate a relevant binocular paracentral scotoma. However, fail–pass criteria for visual field defects are not well established as it is not clear what extent of visual field is necessary for safe driving. In order to avoid unnecessary license denial, some countries, including The Netherlands, offer an “exceptional case” program, mandating an on-road driving test for those candidates who do not quite fulfill the visual field criteria as mentioned above, in which they may demonstrate that they are well adapted to their visual field defects in natural driving conditions.^18^

Entry in this “exceptional case” program is based on visual acuity and visual field testing, generally the binocular Esterman visual field test.^19^ This test was adopted by the American Medical Association for assessment of impairment.^20^ It is a suprathreshold binocular test and includes peripheral points. It is fast and readily available on most commercial visual field testing devices. As a result, it has become the test of choice in The Netherlands and also in the United Kingdom, Norway, Sweden, and Ireland.^21^ However, the Esterman visual field test has several flaws, such as that the distance between test points is not equal and that stimuli are not presented by the principle of the “hill of vision.” The Dutch Society of Ophthalmology (NOG) recommends the use of the Esterman visual field in relation to driving until a tailor-made, well-evaluated screening tool is developed.^22^

Unclear fail–pass criteria for visual field defects might lead to more on-road driving tests than are required on the grounds of the visual field. The on-road driving tests, although providing high-quality discrimination between capable and incapable drivers, are costly and time-consuming. It would be desirable to reduce the number of cumbersome on-road driving tests offered to persons with visual field defects by redefining which “exceptional cases” would benefit from performing an on-road driving test while maintaining (or even improving) the quality of the discrimination.

Concurrently, it is important to give every potential driver the chance to obtain a license and to reduce the number of unjust license withdrawals for individuals with visual field defects. For elderly people, driving a motor vehicle may be the only option for autonomous mobility. Driving cessation in older drivers is related to poorer mental health, such as depression, declines in cognitive abilities, and diminished physical and social functioning.^23^ Individuals who have their driving license withdrawn due to visual field loss also experience welfare loss on several domains, such as work, income, housing, and health.^24^ Additionally, if withdrawals are experienced as unfair by those affected, it might lead to a decreased trust of the authorities, especially if withdrawals are based on vision tests that do not predict individual driving performance very well.^25^

Previous research on the predictive value of the visual field on driving performance was not conclusive. In the study by Silveira et al.,^26^ the Esterman visual field 120-degree criterion could not predict the result of an on-road driving test.^26^ However, the nonsignificant results could have been due to the small proportion of people who failed the on-road driving test and the small number of participants with visual field loss (8/94). Dow^27^ found that failure to meet the formal visual field standards, based on the Esterman visual field, does not impede passing the on-road driving test, and no single factor or combination of factors could predict failure of the road test.^27^ Their study was not balanced: the number of failed candidates was much smaller than the number of passes, and there was no formal assessment between the extent of the visual field defects and the outcome of the on-road driving test. Another study found that the extent and location of visual field loss did not have significant impact on driving performance. However, the extent of the visual field was obtained by confrontation testing, which can be considered less reliable than automated visual field testing.^28^

For this article, the aim is to explore the predictive value of the Esterman visual field in relation to the outcome of the on-road driving test by performing a matched case-control study containing balanced groups for pass and fail outcomes of the on-road driving test. Given the limited value of visual field testing, the question is if any prediction can be made about the results of the on-road driving test (or, for that matter, driving performance in general) based on the outcome of the visual field test. It may be possible to predict in which cases an individual Esterman visual field alone can warrant license renewal and avoid the on-road driving test. Some candidates could have severe visual field defects that predict a failed on-road driving test or, on the other hand, such mild defects that a passed result is expected. The location of the visual field defects could also have a relevant relation to the outcome of the on-road driving test. Possibly, the role of visual field testing, as a tool for preselection into the “exceptional case” program, could be optimized to limit the number of on-road driving tests.

## Methods

### Participants

A retrospective case-control study was performed. Participants were selected from a database provided by the CBR (Dutch driving test organization), the central office for driver's license administration in The Netherlands. The included participants applied for a renewal of their group 1 driving license (categories A, motor; B, passenger car; BE, trailer for passenger car; and T, tractor) and performed an on-road driving test, based on their visual field defects, between May 2010 and June 2015. When an on-road driving test is mandated based on an insufficient visual field, then the binocular (distance) visual acuity must be at least 0.30 (logarithm of the minimum angle of resolution [logMAR]) and the binocular visual field must extend at least 90 degrees horizontally. Candidates with visual field defects with a binocular visual acuity above 0.30 (logMAR), a severely affected central visual field, and/or a minimal horizontal visual field extension below 90 degrees are not considered for an on-road driving test and not granted a license. See Supplementary Material S1 for more information about the driver's license application process in The Netherlands.

All included participants performed an on-road driving test, based on their visual field defects. Included cases were participants who did not pass the on-road driving test. For each case, a control participant was selected who did pass the driving test, matched on age (month of birth versus month of testing), gender, and the diagnosis code for their disorder in the CBR database. The codes referred to “visual field defect” (inclusion criterion for all participants in the database), “progressive eye disease” (e.g., glaucoma), and “central nervous system disorder” (e.g., occipital stroke).

Only participants were included in whom a visual field defect was the main (if not only) reason to mandate the on-road driving test. Furthermore, for inclusion, a binocular Esterman visual field (as provided by the referring ophthalmologist) had to be available. All Esterman visual fields were tested with the custom program available on a Humphrey Visual Field Analyzer (HFA; Carl Zeiss Meditec AG, Jena, Germany).

### The On-Road Driving Test

All participants in this study underwent an on-road driving test on the public road, according to usual clinical practice in The Netherlands, where right-hand traffic and left-hand drive are common practice. The test has a duration of 60 minutes, with a minimal driving time of 30 minutes, and is administered by a practical fitness-to-drive expert of the CBR. There is not an obligatory fixed route for the test, but a specific protocol is in place in the presence of visual field defects (see Supplementary Materials S2). The on-road driving test always leads to a dichotomous pass or fail outcome. However, a passed driving test can be coupled with imposing restrictions, such as mandatory vehicle adaptations, and occasionally additional advice is given on a limited duration of license renewal.

### Data Extraction

For each participant, the 120 points of the binocular Esterman visual field were scored if the point was “seen” (1) or “not seen” (0). The month and year of birth, month and year of the driving test, (best-corrected) visual acuity of right and left eye, and relevant ocular comorbidity were listed (glaucoma, macular disease, cataract, stroke/brain tumor, diabetes mellitus, ocular vascular occlusion). Furthermore, data reported by the ophthalmologist, such as if the participant wore glasses or contact lenses, if the visual field condition was progressive or stable, and presence of diplopia or affected dark adaptation, were included in the database.

### Analysis of Visual Field Data

**Figure 1.**
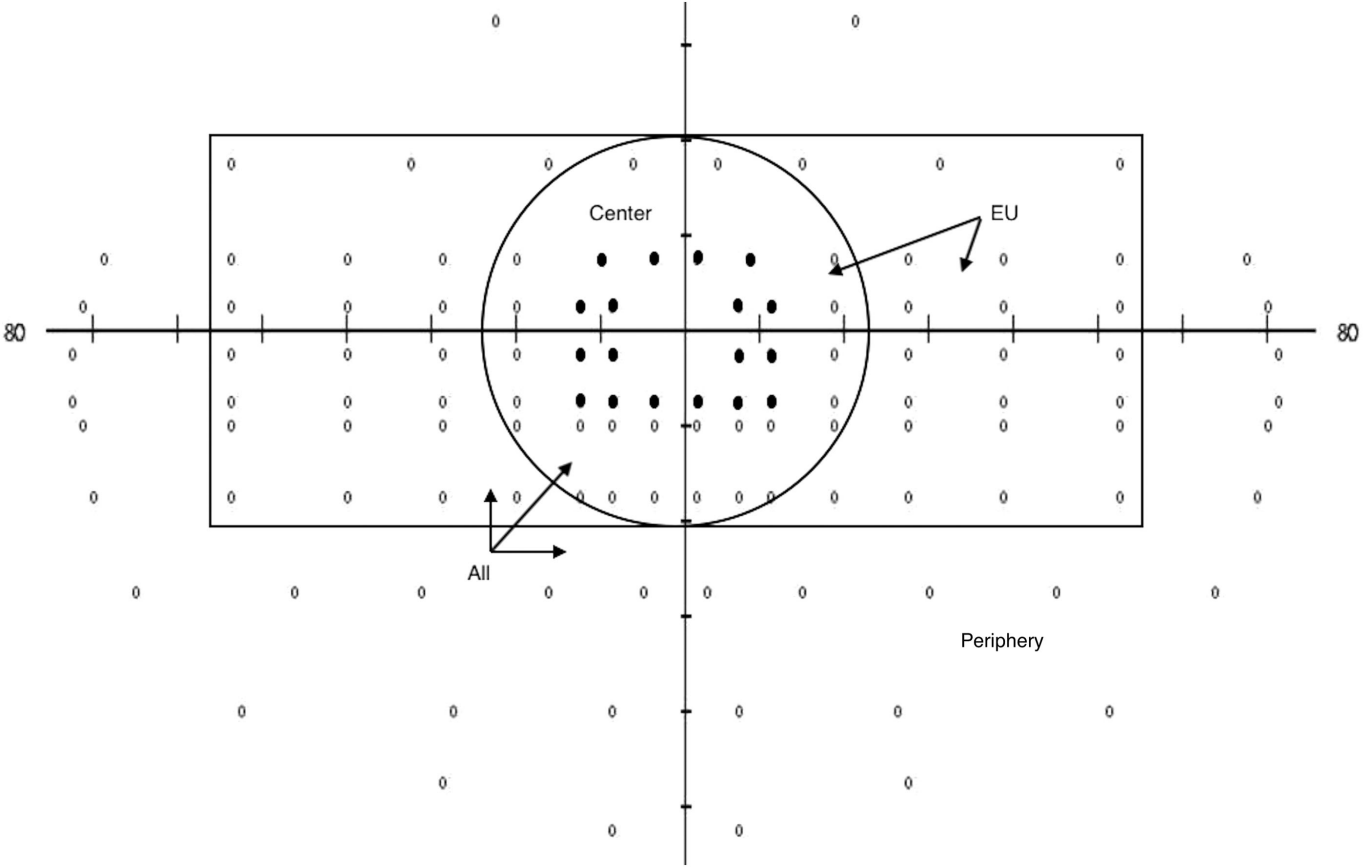
Visual field regions used for analysis. “All”: all 120 points; “center”: all points within 20 degrees (radius) from the center; “EU region”: all points within a rectangle, extending 20 degrees up and down and 50 degrees left and right and “periphery” (the points outside the EU region). The *black dots* represent the paracentral region.

The Esterman visual field was divided in regions for the statistical analysis. These regions were “all” (all points), “center” (points within a 20-degree radius), “European Union (EU) region” (a rectangle extending 20 degrees up and down and 50 degrees left and right, as mandated in the EU regulations^29^), “paracentral” (all 18 points adjacent to the center), and “periphery” (the points outside the EU region) (Fig. 1). Additionally, the visual field was divided by the midlines in “left,” “right,” “up,” and “down” and the quadrants “left-up,” “right-up,” “left-down,” and “right-down.”

### Statistical Analysis

Descriptive statistics were reported for the participant characteristics in the database. In addition, *t*-tests for continuous variables and χ^2^ tests for categorical variables were performed to verify if the matching was performed properly and if the groups did not vary significantly on the reported characteristics.

The relation between the factor (number of points seen) and the dichotomous outcome (fail or pass of the on-road driving test) was tested with a logistic regression model for each of the regions in Figure 1. The assumptions for logistic regression were checked. For skewed distributions, a ln-transformation was used. If this did not normalize the distribution, the variable was divided in tertiles. In case of tied values, the lowest rank was used. Besides conducting an uncorrected model, a corrected model was also performed, including best-corrected visual acuity (visual acuity of the best eye, in logMAR), age, and gender, to each visual field region to accommodate (minor) differences between cases and controls. Each model was also tested for the group of patients with glaucoma—since glaucoma is the main reason for driver's license issues at a higher age, from an ophthalmologic point of view—to investigate if the regions can predict the outcome of the on-road driving test for this group of patients. We decided to use the same tertiles in the group of patients with glaucoma as were used in the models for the total population. Other variables, such as presence of ocular comorbidities and progressive nature, were added to the univariate model of the whole field for the total population as these were potential confounders.

To investigate the predictive value of the Esterman visual field outcome for the outcome of the on-road driving test, receiver operating characteristic (ROC) curves were computed for each of the visual field regions, investigating the relation between the number of points seen and the outcome of the driving test. Pearson correlations for pairs of continuous variables were computed to explore the dependency of neighboring regions.

All analyses were performed with IBM SPSS Statistics for Windows, Version 26.0 (SPSS, Armonk, NY, USA).

This research followed the tenets of the Declaration of Helsinki. Formal approval of an ethical committee was not necessary, because anonymized data were used for this retrospective analysis. A waiver was obtained from the Medical Ethical Committee of Amsterdam University Medical Centers—location VU University Medical Center.

## Results

### Demographic Description of Participants

In total, 101 cases and an equal number of controls were included. The participant characteristics are shown in Table 1, which reveals that the matching process based on age, gender, and diagnosis code was effective. Also, visual acuity, progressive nature, use of prescription glasses/contact lenses, and the presence of disease were not statistically different between fail and pass groups. Multiple comorbidities could be present in one participant. Forty-nine participants had cataract as reported by the referring ophthalmologist; this was in all cases accompanied by another diagnosis that explained the visual field loss. The average period between the Esterman visual field and the on-road driving test was 3.26 ± 2.87 months. This value did differ significantly, with a longer period for cases than for controls. The study population contained mainly elderly participants with a median age of 80.0 years (minimum, 18.4 years; maximum, 93.7 years). Of the 101 candidates who passed the driving test, 14 were given a license for 1 year, 31 for 3 years, 53 for 5 years, 2 for 10 years, and 1 unlimited.

**Table 1.** Participant Characteristics

Characteristic	Fail (*n* = 101)	Pass (*n* = 101)	*P* Value
Age, mean ± SD, y	76.8 ± 12.7	77.9 ± 12.6	0.522
Number of female participants	21	21	1
Corrected visual acuity (logMAR), mean ± SD	0.120 ± 0.114	0.109 ± 0.110	0.503
Visual field defect, *n*	101	101	1
Progressive eye disease, *n*	100	99	0.561
Central nervous system disorder, *n*	16	16	1
Presence of diseases,^a^ *n*			
Glaucoma	60	54	0.395
Macular disease	9	12	0.489
Cataract	26	23	0.622
Stroke/brain tumor	18	13	0.329
Diabetes mellitus	19	18	0.856
Ocular vascular occlusion	5	5	1
Progressive disease, *n*	49	46	0.672
Prescription eyeglasses, *n*	78	76	0.837
Contact lenses, *n*	2	2	1
Affected dark adaptation, *n*	0	0	—
Diplopia, *n*	0	1	0.314
Time between field test and on-road driving test, mean ± SD, mo	3.97 ± 2.45	2.42 ± 1.59	**<0.001**

“Fail” and “pass” reflect the outcome of the on-road driving test. *P* values are outcomes of independent-samples *t*-tests for continuous variables and χ^2^ tests for categorical variables. Bold value highlights a significant value (*P* < 0.05).

a Multiple comorbidities could be present in one participant.

### Relation between Visual Field Defects and Outcome of Driving Test

**Figure 2.**
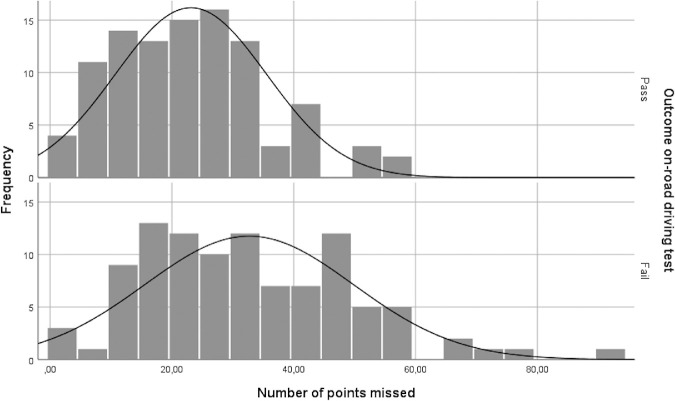
Distributions of the number of points missed for those who passed and failed the on-road driving test. The normal curve is also displayed.

Figure 2 presents the distributions of the number of points missed for the persons with a failed and a passed outcome of the on-road driving test. A wide range of overlap can be seen between the two groups, with the failed group showing a wider and lower normal curve.

Table 2 shows the outcome of the logistic regression analysis for the visual field regions (Fig. 1). A corrected model was also performed, adjusting for age, gender, and visual acuity (visual acuity of the best eye, in logMAR). However the difference with the uncorrected model was not noteworthy; hence, we decided to report the uncorrected model. Most regions presented a positive skew. To correct this, for the regions all, EU, left, right, and down, a ln-transformation was used, and for the other regions, the regression models were conducted with tertiles.

**Table 2.** Number of Points Missed in Visual Field Regions (Mean ± SD) and Odds of a Failed Outcome

	Total Population						Glaucoma Population					
	Fail (*n* = 101)		Pass (*n* = 101)				Fail (*n* = 60)		Pass (*n* = 54)			
Characteristic	Mean ± SD	*n*	Mean ± SD	*n*	Odds Ratio	*P* Value	Mean ± SD	*n*	Mean ± SD	*n*	Odds Ratio	*P* Value
Whole field (120)	32.68 ± 17.15	101	23.13 ± 12.46	101	**2.52 (1.53–4.14)** ^a^	**<0.001**	33.12 ± 17.46	60	23.65 ± 13.22	54	**2.51 (1.30–4.86)** ^a^	**0.006**
EU region (86)	17.32 ± 11.88	101	10.35 ± 6.86	101	**2.45 (1.61–3.73)** ^a^	**<0.001**	17.77 ± 12.16	60	10.72 ± 7.61	54	**2.48 (1.41–4.33)** ^a^	**0.002**
Center (42)												
Tertile 1	0.45 ± 0.51	31	0.31 ± 0.47	45	—	—	0.50 ± 0.52	16	0.26 ± 0.45	19	—	—
Tertile 2	3.41 ± 1.12	27	3.03 ± 1.06	39	1.01 (0.51–1.97)	0.988	3.50 ± 1.02	14	3.17 ± 1.13	24	0.69 (0.27–1.77)	0.442
Tertile 3	11.60 ± 6.29	43	9.18 ± 3.13	17	**3.67 (1.78–7.58)**	**<0.001**	12.27 ± 6.93	30	10.09 ± 3.53	11	**3.24 (1.24–8.45)**	**0.016**
Paracentral (18)												
Tertile 1	0.00 ± 0.00	34	0.00 ± 0.00	58	—	—	0.00 ± 0.00	16	0.00 ± 0.00	28	—	—
Tertile 2	1.41 ± 0.50	27	1.42 ± 0.50	24	1.92 (0.96–3.84)	0.066	1.46 ± 0.52	13	1.50 ± 0.52	12	1.90 (0.70–5.14)	0.208
Tertile 3	5.55 ± 2.52	40	4.11 ± 1.45	19	**3.59 (1.80–7.17)**	**<0.001**	5.55 ± 2.72	31	4.21 ± 1.67	14	**3.88 (1.61–9.35)**	**0.003**
Periphery (34)	15.37 ± 6.88	101	12.78 ± 7.44	101	**1.05 (1.01–1.10)**	**0.013**	15.35 ± 6.46	60	12.93 ± 7.46	54	1.05 (1.00–1.11)	0.068
Left (60)	15.49 ± 10.38	101	10.90 ± 7.72	101	**1.75 (1.22–2.51)** ^a^	**0.002**	14.93 ± 10.43	60	11.85 ± 7.91	54	1.37 (0.85–2.22)^a^	0.198
Right (60)	17.20 ± 12.55	101	12.23 ± 8.63	101	**1.38 (1.02–1.88)** ^a^	**0.039**	18.18 ± 12.34	60	11.80 ± 9.33	54	**1.82 (1.18–2.81)** ^a^	**0.007**
Up (38)	11.29 ± 6.51	101	8.05 ± 4.70	101	**1.11 (1.05–1.17)**	**<0.001**	11.75 ± 6.76	60	7.89 ± 4.69	54	**1.12 (1.05–1.20)**	**0.001**
Down (82)	21.40 ± 13.15	101	15.08 ± 10.36	101	**1.91 (1.30–2.82)** ^a^	**0.001**	21.37 ± 13.72	60	15.76 ± 10.95	54	**1.76 (1.06–2.92)** ^a^	**0.030**
Left-up quadrant (19)												
Tertile 1	1.61 ± 1.18	36	1.49 ± 1.19	49	—	—	1.60 ± 1.14	20	1.71 ± 1.20	24	—	—
Tertile 2	4.88 ± 0.78	33	4.88 ± 0.84	34	1.32 (0.69–2.52)	0.397	4.75 ± 0.79	20	4.83 ± 0.86	18	1.33 (0.56–3.18)	0.517
Tertile 3	10.41 ± 2.95	32	8.67 ± 1.88	18	**2.42 (1.18–4.97)**	**0.016**	10.50 ± 2.78	20	8.50 ± 2.02	12	2.00 (0.79–5.07)	0.144
Right-up quadrant (19)												
Tertile 1	1.46 ± 1.15	35	1.40 ± 1.23	45	—	—	1.63 ± 1.21	19	1.44 ± 1.25	27	—	—
Tertile 2	4.88 ± 0.83	25	4.81 ± 0.79	36	0.89 (0.46–1.75)	0.742	4.88 ± 0.89	16	4.72 ± 0.75	18	1.26 (0.52–3.09)	0.608
Tertile 3	10.12 ± 2.82	41	9.10 ± 2.00	20	**2.64 (1.32–5.27)**	**0.006**	10.36 ± 2.90	25	8.00 ± 1.00	9	**3.95 (1.51–10.33)**	**0.005**
Left-down quadrant (41)												
Tertile 1	2.15 ± 1.51	27	1.45 ± 1.25	42	—	—	2.11 ± 1.45	18	1.63 ± 1.34	19	—	—
Tertile 2	7.09 ± 1.79	35	7.67 ± 2.15	36	1.51 (0.77–2.96)	0.227	6.86 ± 1.62	21	7.61 ± 2.10	23	0.96 (0.40–2.31)	0.934
Tertile 3	18.10 ± 5.67	39	16.04 ± 4.20	23	**2.64 (1.30–5.35)**	**0.007**	17.95 ± 6.36	21	17.00 ± 5.27	12	1.85 (0.71–4.82)	0.210
Right-down quadrant (41)												
Tertile 1	2.03 ± 1.32	35	1.59 ± 1.46	37	—	—	2.37 ± 1.26	19	1.38 ± 1.53	21	—	—
Tertile 2	7.72 ± 2.49	25	8.05 ± 2.00	41	0.65 (0.33–1.27)	0.205	7.67 ± 2.41	15	7.55 ± 1.85	20	0.83 (0.33–2.07)	0.687
Tertile 3	21.59 ± 5.86	41	18.61 ± 5.36	23	1.88 (0.95–3.75)	0.071	21.65 ± 6.83	26	20.08 ± 5.33	13	2.21 (0.89–5.49)	0.088

The total number of points behind the field region demonstrates the number of points tested in the regions as defined in Figure 1. “Fail” and “pass” reflect the outcome of the on-road driving test. Odds ratios and *P* values are outcomes of univariate logistic regression models using untransformed variables, ln-transformed variables, or dummy variables. The second model only included the participants with a glaucoma diagnosis. Tertile 1 was used as the reference group in the logistic regression models. Bold values highlight significant values (*P* < 0.05).

a Odds ratio is computed with ln-transformed number of points missed. Mean ± SD is presented as untransformed variable.

The logistic regressions show that for most visual field regions, there was a significant increased odds for not passing the driving test when more points were missed. The relation was somewhat stronger for the center of the visual field, and the left side had more significant outcomes than the right side.

The logistic regression analysis for the group with a glaucoma diagnosis similarly showed that for the whole field, EU region, and center and paracentral regions, a significant increased odds for not passing the driving test was found when more points were missed. The visual field regions, right, up, down, and right-up quadrant, showed a significant relation, which were regions in the center and the right side of the visual field.

The analyses of additional variables showed that only the time between the Esterman visual field based on the whole field data and the on-road driving test was a significant confounder and slightly increased the odds of a failed outcome (2.61; 95% confidence interval [CI], 1.53–4.47; *P* < 0.001).

### Predictive Value of the Visual Field Outcome for the Outcome of the Driving Test

**Figure 3.**
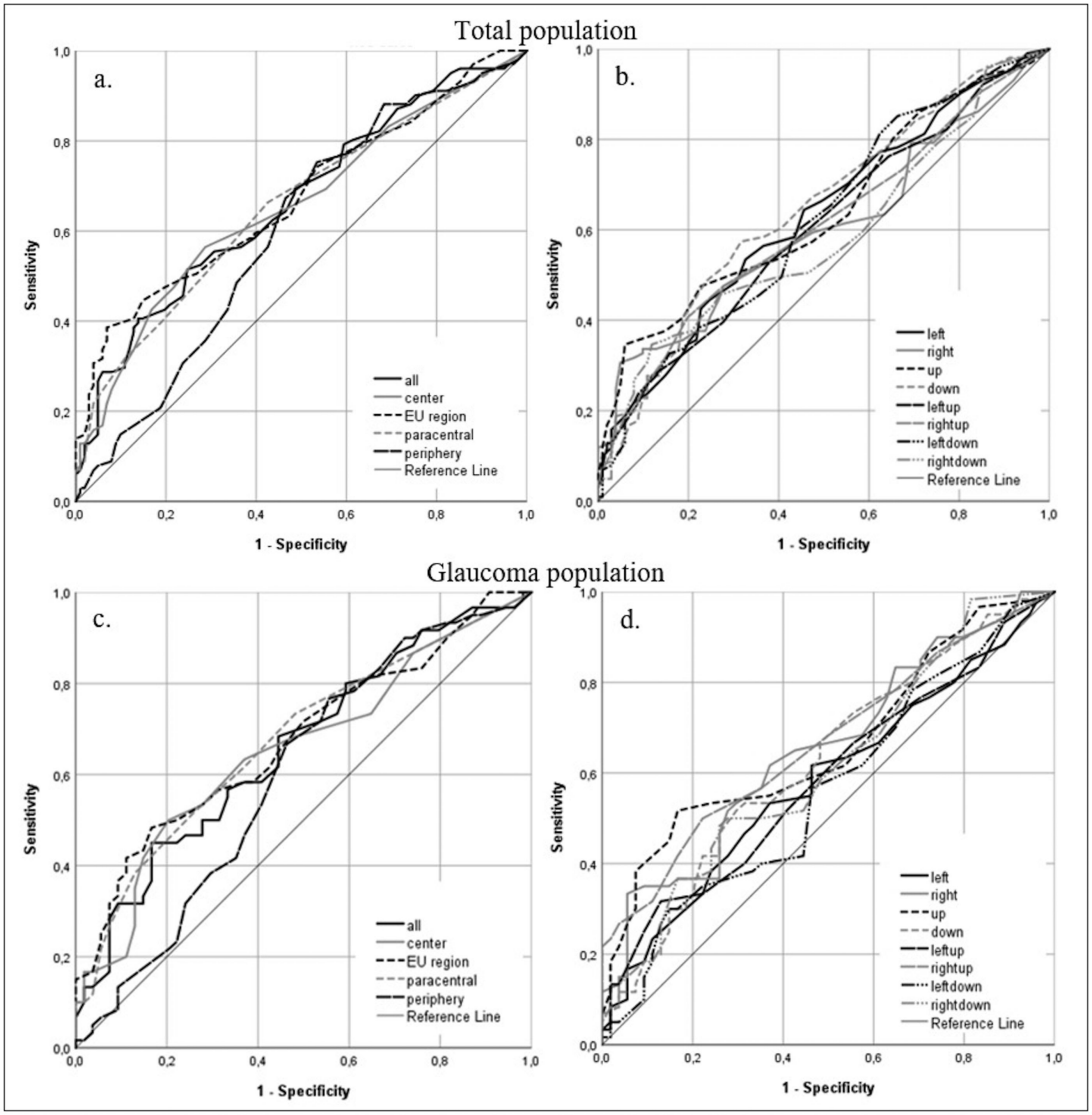
ROC curves for the relation between number of points seen per visual field region and outcome of on-road driving test for the total population (a, b) and the glaucoma population (c, d). (a) The area under the curve was 0.664 (whole field), 0.655 (central 20 degrees), 0.674 (EU region), 0.655 (paracentral points), and 0.601 (periphery). (b) The area under the curve varied between 0.585 (right-down) and 0.648 (down). (c) The area under the curve was 0.659 (whole field), 0.655 (central 20 degrees), 0.676 (EU region), 0.675 (paracentral points), and 0.598 (periphery). (d) The area under the curve varied between 0.560 (left-down) and 0.665 (right-up).

Figure 3 shows the ROC curves for the relation between a failed outcome for the on-road driving test and the number of points seen in the visual field regions. For all the regions, the predictive value, as measured by the AUC of the ROC curve, was below 0.7, which indicates no discriminative ability for the outcome of the test.^30^ The EU region had the highest predictive value for the total population (0.674) and the glaucoma population (0.676). This implies that the results of the Esterman visual field test are not suitable for distinguishing between participants who will fail or pass the on-road driving test in our population.

Table 3 shows the intracorrelations between the visual field regions. It shows that the number of points missed is independent of neighboring regions, as the correlations between nonoverlapping visual field regions were well below 0.7.

**Table 3.** Intracorrelations for the Visual Field Regions

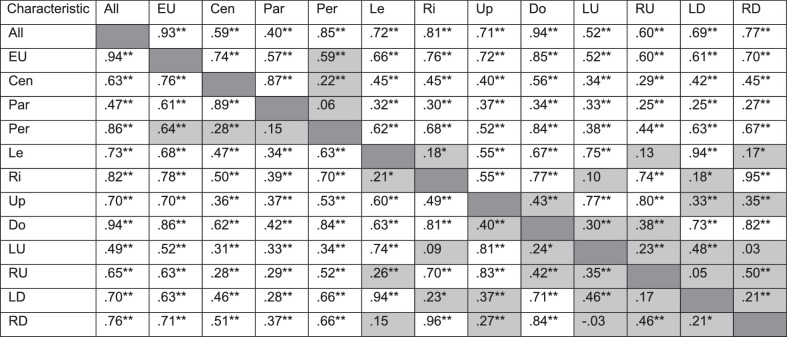													

Above the diagonal: total population (*n* = 202); below the diagonal: glaucoma population (*n* = 114). Pearson correlations for pairs of continuous variables. Shaded cells represent correlations between nonoverlapping regions. Cen, center; Par, paracentral; Per, periphery; Le, left; Ri, right; Do, down; LU, left-up; RU, right-up; LD, left-down; RD, right-down.

* *P* < 0.05.

** *P* < 0.01.

## Discussion

In this research, we investigated the relation between the Esterman visual field test and the outcome of the on-road driving test. We found that, for most regions of the visual field, there is a significantly increased odds for failing the driving test when more points are missed. This underlines the importance of the visual field for driving performance. We found that the center may be more important than the periphery and that the left side may be more important than the right side. This could be attributed to the practice of right-hand traffic and left-hand drive in The Netherlands. It is also consistent with current opinions about the relative importance of the various visual field regions.^4^

We found that the period between the Esterman visual field and the on-road driving test was approximately 1.5 months longer for cases than for controls, with a wider standard deviation for cases. A probable explanation is that a failed on-road driving test is often followed by a second on-road driving test, after the candidate takes additional driving lessons. We used data of the driving test that defined the candidate as suitable or unsuitable; hence, the time between the visual field test and the on-road driving test is longer for cases than for controls. The wide standard deviation can be explained by a variety in amount of driving lessons between the two failed on-road driving tests. We had no access to data about the number of failed on-road driving tests and the number of driving lessons.

The predictive value of visual field defects for the outcome of the driving test provided poor results. There was a large overlap between the visual field outcomes of failed and passed driving tests (see Fig. 2), making it virtually impossible to predict the outcome of the driving test, based on the visual field test alone. The poor predictive value illustrates that laboratory visual field testing and on-road driving tests measure different entities/capacities of the visual (visual field) and motor and cognitive (on-road driving test) system.

The Esterman visual field test was adopted for the assessment of impairment,^20^ for which the central part of the field is considered more valuable than the periphery, the lower hemisphere is more useful than the upper, and the central 10 degrees is not measured. However, this is not necessarily applicable for driving. Typically, most traffic events occur within the central 25 degrees of the visual field. The front windshield of a car usually allows for visual field extension to the left of about 20 degrees and 50 degrees to the right. Side windows and mirrors are located more into the periphery. Moreover, the stimuli of the Esterman visual field do not follow the “hill of vision” but have size III and an intensity of 10 dB. This is far above the suprathreshold in the center of the visual field.^22^ A new traffic perimetry test has recently been proposed,^21^ removing some of the limitations of the Esterman visual field, such as emphasizing all parts of the field equally, equidistant test points, and following the hill of vision. The question remains if a static test would be able to predict the outcome of the on-road driving test. An alternative approach allowing a free gaze, with dynamic stimuli and cognitive screening, could be useful in minimizing the cumbersome on-road driving tests.

To the best of our knowledge, this is the first study to examine the relationship of the Esterman visual field with the outcome of an on-road driving test in a systematic manner using balanced and matched groups for the fail and pass outcome. The use of various subdivisions of the Esterman visual field and statistical analysis with odds ratios and ROC curves provides a comprehensive exploration of the research question.

This study has several limitations. The number of participants in this study (*n* = 202) was acceptable for the statistical tests conducted. However, the group of patients with glaucoma (*n* = 114) is a smaller sample size, probably resulting in more nonsignificant results.

We assume the on-road driving test as the gold standard for driving performance. Although this is generally accepted, it may be debated.^31^ The expert on practical fitness to drive who administered the on-road driving test was not blinded to the candidate's diagnoses and visual field defects; otherwise, planning a suitable on-road driving test is not possible. This could have biased the outcome of the on-road driving test. It has been found that driver status (active versus inactive driver) was a predictor for on-road driving performance.^17^ In our retrospective study, we had no data on driver status or experience. Also, information on cognitive function, former rehabilitation, and the time since defect was missing in the provided data, which could have influenced our data and been attributed to a better predictive ability of our model.

Since evenly distributed missed points might have a different impact on driving capacity than clustered missed points, ideally, a cluster analysis should be performed. In a preliminary analysis, we performed a grouped point analysis. We demonstrated that the “weight” of a missed point increased somewhat when there was another missed point at (optimal) 0.3 radians distance. However, the analysis was hampered by the uneven distribution of distances between points in the Esterman visual field. Moreover, the discriminative ability of the test improved only minimally.

A retrospective case-control study was conducted, consequently having no strict control over the inclusion into the “exceptional case” program or insight into the decision of the medical advisor of the CBR. We had access only to candidates who were offered an on-road driving test in the “exceptional case” program. This excludes data on the candidates who were granted a license without administering a driving test and the candidates who were denied entry in the “exceptional case” program and had their license withdrawn without administering a driving test.

## Conclusion

The results of this study confirm the relation between visual field damage and impaired driving performance. When more points are seen, the likelihood of passing the on-road driving test increases. However, in our group, the Esterman visual field test shows no discriminative ability to predict driving performance on an individual level. This implies that, in our group with moderate visual field defects, the number of on-road driving tests cannot be further reduced by a more detailed definition of fail–pass criteria, based on the Esterman visual field test alone. Hence, no adjustments to policy can be made to reduce the number of tests based on our results. This study underlines the need of an accessible and reliable test that can better predict the outcome of the on-road driving test in order to regulate entry in the “exceptional case” program. Such an alternative test may, ideally, combine the advantages of the visual field test (standardized, cheap) with those of the on-road driving test (natural, dynamic stimuli, allow for free gaze movements). Up until now, such a test is not available.

## Supplementary Material

Supplement 1
