# Supplementary material for: Predictive Value of the Esterman Visual Field Test on the Outcome of the On-Road Driving Test
Source: Transl Vis Sci Technol. 2022 Mar 17;11(3):20. doi: 10.1167/tvst.11.3.20 (PMC8944391; doi:10.1167/tvst.11.3.20)
Supplement: Supplement 1 [file tvst-11-3-20_s001.pdf]

Supplementary material

S1 Medical driving license application process in The Netherlands

In The Netherlands, a declaration of health must be submitted when a subject applies for a driving license for the first time, is aged 75, a driver's license has been given for a limited period and an intermediate notification has been received. They must submit an official statement with questions about their general health. If the subject indicates that there is an eye disorder, restricted vision, previous eye surgery or (previous) treatment by an ophthalmologist, then a formal assessment by an ophthalmologist is mandated. The ophthalmologist assesses visual acuity and (by the confrontation method) the visual field. Also, media and fundi are examined. If there is any doubt regarding the visual field, based on the confrontation results, the medical history or the results of the ophthalmological examination, a binocular visual field test (generally Esterman) is mandatory. If there is any sign of double vision or impaired night vision then additional examinations are requested. The ophthalmologist reports the findings regarding visual acuity, visual field, double vision, night vision, cause of any impairments and if the impairments are stable or progressive in nature. Also, an advice is included regarding the validity of the license (group 1 or 2, not fit to drive, fit to drive or fit to drive for a limited period: 1, 2, 3, 5 or 10 years). It can be advised that the license is only granted after an on-road driving test is performed.

The medical advisor of the CBR (a medical doctor) makes the decision about the administration of the license, the period of validity, special restrictions (e.g. daytime only) and whether or not a driving test is mandated.

S2 The on-road driving test

All participants in this study underwent an on-road driving test in The Netherlands, where right-hand traffic and left-hand drive are common practice. This test is administered by an expert on practical fitness to drive of the CBR. In principle the test takes place in the participant's own vehicle on the public road. A driver training vehicle is chosen in case of (hemi)anopsia, long refrainment from driving, after a period of driving lessons due to a previous failed on-road driving test and if other vehicle adaptations are required. The duration of the test is 60 minutes with a minimal driving time of 30 minutes. There is not an obligatory fixed route for the test. However, the CBR has designed a specific protocol for on-road driving tests administered in the presence of visual field defects. This protocol includes the test must always contain a few left/right turns, different types of intersections (both straight on and left turns), roundabouts (both ½ and ¾ rounds), roads outside urban areas (driving with higher speeds, overtaking or passing slower vehicles, driving on a winding road), highways (changing lanes, overtaking other vehicles, choosing direction independently and rounding long bends at high speed with correct position) and narrow crowded roads with bicycle traffic.

The scoring system of the TRIP (Test Ride for Investigating Practical fitness to drive) is used.[1] The on-road driving test always leads to a dichotomous pass or fail outcome. However, a passed driving test can be coupled with imposing restrictions, such as mandatory vehicle adaptations, and occasionally additional advice is given on a limited duration of license renewal.

1. Tant MLM. *Visual performance in homonymous hemianopia: assessment, training and driving*: Groningen, The Netherlands: University Library Groningen; 2002.
